# Effects of binaural and monaural beat stimulation on attention and EEG

**DOI:** 10.1007/s00221-021-06155-z

**Published:** 2021-07-10

**Authors:** Hessel Engelbregt, Marinda Barmentlo, Daniel Keeser, Oliver Pogarell, Jan Berend Deijen

**Affiliations:** 1 Hersencentrum Mental Health Institute, Amsterdam, the Netherlands; 2 Department of Psychiatry and Psychotherapy, Ludwig-Maximilians-University of Munich, Munich, Germany; 3 Faculty of Behavioural and Movement Sciences, Section Clinical Neuropsychology, Vrije Universiteit, van der Boechorststraat 7, 1081 BT Amsterdam, the Netherlands

**Keywords:** Attention, Flanker task, Binaural beats, Monaural beats, Auditory beat stimulation, EEG

## Abstract

Nowadays a popular technique to improve mood and cognition is auditory beat stimulation (ABS), which is thought to induce a frequency-following response of brainwaves. The main types of ABS are monaural beats (MB) and binaural beats (BB). BB involves the presentation of a specific frequency to one ear and another frequency to the other ear which may induce neural entrainment. A difference between the frequencies of 40 Hz is assumed to improve cognition. The present study examined the effect of 40 Hz binaural beats (BB) and monaural beats (MB) on attention and electroencephalography (EEG). A total of 25 first-year psychology students (11 males, 14 females) performed a Flanker task while EEG was recorded during the 5 min-presentation of pink noise (PN), MB and BB. With respect to attention, as measured by the Flanker task, the number of false responses in the BB condition was smaller than that in the PN condition while the number of false responses in the MB condition was larger as compared to the PN condition. As there was no association of BB with a consistent increase in absolute 40 or 45 Hz power compared to PN or MB, EEG recordings could not confirm the hypothesized neural entrainment in the brain. Overall, the current findings show that listening to 40 Hz BB improves attention but do not show the occurrence of neural entrainment. Future research is recommended to include a larger sample, to use a broader cognitive test battery and to present auditory beats with a longer duration.

## Introduction

Nowadays, our competitive society is characterized by a lot stress due to the digital overload of information from desktops, laptops, tablets, and smartphones. All day and even night, people are bombarded with so many messages and alerts that focussing becomes increasingly harder and nearly impossible. As a consequence, people are searching for methods to unwind and get relaxed. In addition to the increasingly used method for relaxation of mindfulness meditation, a popular technique to improve mood and cognition is auditory beat stimulation (ABS), which is thought to induce a frequency-following response of brainwaves associated with an improvement of mood and cognition. The main forms of ABS are binaural beats (BB) and monaural beats (MB), which are both characterized as two simultaneously presented sine waves with a stable amplitude and slightly different frequencies (Chaieb et al. 2015). Although two tones are presented, subjects hear them as one tone, at the frequency of the difference between these two inputs. For example, when two tones of 200 and 240 Hz are presented, the subject perceives a 40 Hz beat. These auditory stimuli are called monaural beats when they are both presented to each ear and binaural beats when the one tone is presented to one ear and the other tone separately to the other ear. In response to BB, hearing nerves in the brain send the sound to the superior olivary nucleus before it reaches the cortex, where a tone is consciously perceived (Kasprzak 2011). In the superior olivary complex neurons are especially sensitive to phase shifts between both ears and will thus fire in a rate corresponding to the phase difference of the BB (Chaieb et al. 2015). The phase difference of the BB will eventually lead to neural entrainment in the brain. This means that a neural resonance, i.e., neural synchronization, may occur as the eventual consequence of the auditory stimuli (Large et al. 2015). The manifestation of neural entrainment has been supported by the results of electrophysiological studies. For instance, steady-state evoked potential (SS-EP) studies found that a periodic rhythm produced a sustained response in the delta (2.4 Hz) band (Nozaradan et al. 2012a) and complex rhythms were found to elicit multiple SS-EPs at frequencies in the electroencephalography (EEG) corresponding to the rhythmic pattern envelope, while the selectively enhanced SS-EPs amplitude at pulse suggests the occurrence of neural oscillations (Nozaradan et al. 2012b).

A number of studies using magnetoencephalography (MEG) and EEG confirmed that fluctuations in delta (2.4 Hz), beta (20–37 Hz) and gamma (40 Hz) band power synchronize with intermittent and metrical auditory rhythms (Fujioka et al. 2009; Nozaradan 2014; Schneider et al. 2002). With respect to specifically the gamma band, rhythmic tone sequences have been found to evoke short-latency gamma-band (20–60 Hz) activity which diminished during tone omissions (Snyder and Large 2005).

In spite of the above evidence of neural entrainment, it is not fully confirmed yet that BB leads to a frequency following response in the brain. To examine the specific effect of BB on EEG recordings Gao (2014) presented BB in delta (1 Hz), theta (5 Hz), alpha (10 Hz), and beta (20 Hz) band frequencies. Healthy participants listened to each type of BB for 5 min at a time, with 2-min breaks in between, while EEG was recorded. Not any type of the BB elicited clear brain entrainment in the relative power of EEG. During delta and alpha BB, the relative power of theta and alpha increased and that of beta decreased, while during theta BB the relative power of beta decreased and during beta BB the relative power of theta decreased. The authors conclude that there was no frequency following the response of the presented BB. In a similar study BB were created by adding a sinewave, differing in 4.53 Hz, (theta-beat), 8.97 Hz (alpha-beat), 17.93 Hz (beta-beat), 34.49 Hz (gamma-beat) or 57.3 Hz (upper gamma-beat), to a pure tone of 373 Hz in all conditions. Results indicated no significant effects of BB for any of the beat frequencies within the corresponding EEG bands (López-Caballero and Escera 2017). Based on the number of oscillations per second these BB-induced phase differences can be categorized into different clusters which are associated with specific brain functions. As a consequence, the effects of BB on brain activity are thought to be dependent on increasing the power of particular frequency bands. For instance, delta frequency (0.5–4 Hz) BB may increase delta band power and induce its associated deep sleep. Similarly, theta (4–8 Hz) BB may increase theta power and its associated state of deep relaxation, alpha BB (8–13 Hz) the power of alpha band which is associated with a quiet and relaxed attention, beta BB (13–40 Hz) beta band power and its associated thinking, concentrating and processing information and, finally, gamma BB (40 Hz and higher) gamma-band power and its associated attentional selection, memory, associative learning and positive emotional feelings (Teplan 2002). A number of studies of BB inducing these different frequencies have yielded a diversity of results.

For instance, it has been found that alpha (9.55 Hz) improves working memory (Kraus and Porubanová 2015) and beta frequency (16 and 24 Hz) BB improves the performance on a number vigilance task (Lane et al. 1998). In addition, anxiety was found to decrease in people after listening to delta (0–4 Hz) frequency BB for 60 days (Wahbeh et al. 2007). With respect to gamma BB, in a study by Reedijk et al. (2015) participants listened to gamma (40 Hz) BB, alpha (10 Hz) BB or a constant tone of 340 Hz as control condition before they performed an attentional blink task. Results indicated that gamma BB, but not alpha BB, reduced the attentional blink in individuals with low striatal dopamine, using spontaneous eye blink rates as a marker of the individual striatal dopamine level. The authors conclude that gamma BB led to increase of divergent thinking, reflecting more cognitive flexibility. A subsequent study investigated whether gamma BB increases the flexibility of cognitive-control style. Participants were presented with gamma (40 Hz) BB or a constant tone of 340 Hz (control condition) during 3 min before performing a dual task. After exposure to gamma BB a more pronounced response-compatibility effect, reflecting distributed parallel processing, was found supporting the assumption that gamma BB promotes cognitive flexibility (Hommel et al. 2016). In a slightly more recent study examining attentional focusing healthy adults listened for 3 min before and during a global–local task to gamma-frequency (40 Hz) binaural beats based on a 340 Hz carrier tone, which was used as constant tone in the control condition. Results indicated that visual attention became more focused after gamma frequency BB compared to the control condition, suggesting that gamma BB may enhance the focus of attention (Colzato et al. 2017). Recently, the effects of 40 Hz gamma BB and 40 Hz gamma MB on attention and working memory in healthy adults were compared with those of white noise. As speed of performance of the Flanker attention task was faster under the BB and MB condition than under WN, it appeared that high-frequency BB as well as MB may increase the efficiency of attention processing (Engelbregt et al. 2019).

The present study was aimed to examine whether high-frequency auditory beats would improve attention and induce neural synchronization.

We presented frequencies of 440 Hz and 480 Hz to induce gamma BB of 40 Hz. These frequencies are not fully in line with the notion that BB are perceived when the tone frequencies are approximately between 100 and 400 Hz with an upper-frequency limit for the perception of BB of about 1000 Hz and with differences of no more than 35 Hz (Licklider et al. 1950). However, significant beat frequency responses have been found after the presentation of 40 Hz BB induced by a pair of 380 and 420 Hz (Schwarz and Taylor 2005). Comparably, a more recent study in young adults (21–29 years) used primary tones of 390 and 430 Hz, and 810 and 850 Hz, both pairs inducing a BB rate of 40 Hz. Results indicated that frequency-following responses were elicited by tones in both the lower-frequency range of 390 and 430-Hz and, to a lesser extent, by tones in the higher frequency range of 810 to 850 Hz (Grose and Mamo 2012).

With respect to the measurement of attention, an adapted version of the Eriksen Flanker task was used. This task measures the ability to suppress responses that are inappropriate in a particular context and is categorized as a selective attention task (Eriksen and Eriksen 1974). When subjects perform the Eriksen Flanker Task, a frontal brain structure, the anterior cingulate cortex (ACC), is activated, being more active in response to processing incongruent stimuli than congruent stimuli (Davelaar 2013). In addition, a meta-analysis of neuroimaging studies indicated increased activation in the right dorsolateral prefrontal cortex and the right insula (which separates the frontal and temporal lobe) during the performance of the Flanker task (Nee et al. 2007). In addition to these studies on the Flanker task, it has been found that the capacity to shift attention to new stimuli is mediated by the right temporoparietal junction (rTPJ). In particular, the anterior part of rTPJ is activated during attentional shifting (Krall 2015). Further, functional magnetic resonance imaging (fMRI) revealed that the posterior inferotemporal cortex is involved in attentional control, in addition to parietal and frontal areas (Stemmann and Freiwald 2019).

In accordance with the results of studies measuring gamma-band power we selected relevant electrode locations. For instance, healthy participants receiving 40 Hz-transcranial alternating current stimulation (tACS) over the right temporal lobe showed increased spectral power derived from electrodes T7 and T8 (i.e., T3 and T4 of the International 10/20 system) in the low-mid gamma band (i.e., 30–45 Hz) (Santarnecchi, 2019). Auditory stimulation of 40 Hz) in the right auditory canal of healthy participants induced the largest 40 Hz power spectrum increase at the F3 electrode, contralateral to the stimulated side (Pastor et al. 2002). Van Deursen et al. (2008) evaluated gamma-band oscillations as a diagnostic biomarker in Alzheimer’s disease (AD) and mild cognitive impairment (MCI). The effects of resting state, music listening, story listening and visual stimulation in AD patients, MCI patients and healthy controls were compared. In the subject group as a whole and compared to resting state, music listening increased gamma-band power at electrode locations Fz, F3, F4, F7, F8, Cz, T4, T5 and T6, story listening at F3, F4, F5, F7, F8, Fp1, T5 andT6, and the visual task at F3, F4, F5, F7, F8, Fp1, T5 and T6. From these particular electrode locations, we selected F3, F4, F7, F8, Fz, Pp1, T3, T4, T5 and T6, while Fp2 was added to check for ocular artifacts.

Based on above-cited findings, we expected that in particular BB would improve the performance on the attention task. With respect to EEG recordings, we expected that BB and to a lesser degree MB stimulation would increase the power of EEG lower gamma frequencies. As there is no clear evidence of the precise location in the frontal, temporal and parietal areas where gamma BB-induced EEG spectral power increase can be expected, we could not hypothesize effects on more specific electrode locations than the selected ones.

## Materials and methods

### Participants

The study sample consisted of 25 first-year psychology students between 18 and 28 years of the Vrije Universiteit Amsterdam, the Netherlands (11 males, 14 females; mean age 21.8 years (*SD* = 2.5). Participants were recruited by means of an online student pool (i.e., vu.sona-systems.com) and were rewarded with credit points. As this student pool consisted of psychology students, the recruitment of particularly psychology students was for practical reasons. Being at the start of their study, they could be assumed to represent the population of young adults A priori exclusion criteria were attention deficit (hyperactivity) disorder (AD(H)D), hearing problems and physical disorders that could interfere with EEG measurements (e.g., epilepsy) or for which EEG could pose a health risk.

## Materials

### Attention task

Attention was measured by means of an adapted version of the Eriksen Flanker Task (Eriksen and Eriksen 1974). The task was programmed (programming languages Objective-C and Swift) by the IT department of the Vrije Universiteit. Arrows, instead of letters as in the original task, were used as target stimuli. The test was presented on an iPad and included three series with increasing difficulty levels. Prior to the start of each series, written instructions were presented on the screen, followed by a practice trial. There are two trial types, with either congruent or incongruent stimuli. The congruent trial is a horizontally arranged array of arrows presented in the same direction (e.g., <  <  <  <  < or >  >  >  > >). The incongruent trial has a similar array of arrows, but the middle arrow, the target, is displayed in the opposite direction (e.g., <  <  >  <  < or >  >  <  > >). The first series consisted of trials presenting five green arrows. Participants had to touch, as fast as possible, one of the two arrow-shaped buttons on the bottom of the screen, that pointed in the same direction as the middle arrow. In the next series trials of five red arrows were presented. This time participants were instructed to touch the button that pointed in the opposite direction of the middle arrow. The third series consisted of trials of green or red arrows. If the arrows were green, participants had to touch the button that pointed in the same direction as the middle arrow, and if the arrows were red, they had to touch the button that pointed in the opposite direction of the middle arrow.

All trials started with a fixation cross on the center of the screen for 1000, 1500 or 2000 ms (random), followed by the presentation of five arrows during 2500 ms. The interstimulus interval (ISI) was 100 ms, and maximum response time 2500. In each series 60 trials were presented, with a randomized order of congruent and incongruent trials. The total task duration was approximately 3.5 min. The reaction time (RT) and the number of false responses on incongruent trials of the third series were taken as output variables.

### EEG recording

EEG was recorded using 19-channel electrode caps with international 10–20 electrodes placement (Jaspers 1958) on a 32-channel Deymed system (sampling rate 1024 Hz downsampled to 128 Hz, Notch filter 50/60 Hz, anti-aliasing filter 50 Hz, Butterworth filter 0.1–100 Hz). Electrode skin impedance was kept below 8 kΩ. An electrode at Fpz served as ground electrode. In addition, electrodes were placed on the left and on the right earlobes which were used for offline linked-ear (LE) reference. The EEG system was connected to a portable computer. For each electrode, the absolute power was recorded in μV2.

### Auditory stimuli

Pink noise (PN), monaural beats and binaural beats were presented with a comfortable speech volume through headphones (Sennheiser) connected to an iPod. The headphones were wired through the tubes of a stethoscope to prevent any influence on the EEG recording.

MB as well as BB were presented with frequencies of 440 Hz and 480 Hz, resulting in a perceived frequency of 40 Hz. With respect to MB, both frequencies were transmitted through both channels, whereas the BB 440 Hz was transmitted through one channel and 480 Hz through the other channel. The auditory stimuli were programmed by means of the audio editor Audacity (V2.3) by the IT department of the Vrije Universiteit.

### Procedure

The study took place in a sound-attenuated room at the Vrije Universiteit. Before the start of the experiment the participants received information and signed an informed consent. They were seated in a comfortable chair and were instructed to sit quiet and relaxed and to look forward at the clean wall. Besides eye blinking, no movements were allowed. After the electrode cap was placed, participant number, age and gender were entered in the tablet and the participant started with the practice trials of the Flanker task. Thereafter, participants put on the headphones and auditory volume was set to a level that the participant indicated as comfortable. Subsequently, one of the three conditions started. The order of presentation of PN, BB and MB was randomized, using a within-subject crossover design, meaning that all participants performed the Flanker task during PN, BB and MB. The exposure to PN, MB and BB was 5 min. Conditions were separated by a 1 min break. The parameters of the Flanker task were randomly predefined, which can be assumed to minimize learning effects. During the whole test procedure, the EEG was recorded. The start and end of each auditory condition were indicated by marks on the sampled EEG signals. The total test procedure took about 1.5 h. After the last condition was finished, the EEG cap was taken off and the participants received a debriefing.

This study was positively assessed by the Scientific and Ethical Review Committee of the Faculty of Behavioural and Movement Sciences of the Vrije Universiteit.

### EEG processing

Selection of artifact-free EEG data for further analysis was done by an EEG expert after screening for seizure activity and/or abnormal EEG patterns. Data files were screened for eye blinks, eye-movement in vertical and lateral ways, technical flaws and distortion by frontal and temporally located muscle contractions. For this aspect, the EEG expert visually inspected the EEG data and additionally used the program Persyst 14 (Persyst Development Corporation, San Diego) with the automated—built in tool—for spike analysis. The Persyst spike algorithm allows the detector to be extremely sensitive while maintaining a low false-positive rate and was found to perform similar to human EEG readers. The algorithm uses a set of advanced neural networks, applied across several different montages, to monitor EEG background, the presence or absence of artifacts, the waveform morphology and voltage field spread of possible abnormalities. A more detailed description of the algorithm and comparison with the performance of human EEG readers can be found in Scheuer et al. (2017). For artifact rejection, the automated selection tool of another program (i.e., NeuroGuide (V3.0.0.1)) was used. For ocular artifact rejection electrodes Fp1 and Fp2 were used. Default for eye movement and drowsiness selection is 'high' which is the most sensitive setting and 1.5 standard deviations threshold for the Amplitude Multiplier. The Z Score of 1.5 standard-deviations means that if at least one second of successive instantaneous Z Scores are equal to or less than 1.5 standard deviations then a selection is made (Applied Neuroscience 2018). Data of individual EEG recordings were included only when there was a minimum of 20 s artifact-free data.

### Statistical analysis

The mean reaction time (RT) and number of false responses on incongruent trials of the third series of the Flanker task were used to measure the effect of the different conditions on speed and quality of attention performance. The data were explored to check for normality. All variables deviated from a normal distribution. After square root transformation the data of the false responses appeared to be normally distributed. Therefore, the number of false responses were analyzed by means of mixed Anova, with gender as between subjects factor and condition (e.g., PN, MB and BB) as repeated measures factor. As different gender appeared to influence RT of the Flanker task, we included gender as between subjects factor. To correct for possible baseline differences between males and females, data of false responses in the PN condition served as covariate. As planned comparisons, we used simple contrasts to compare the false responses of the MB condition and the BB condition with those of the PN condition. In spite of square root, log or log 10 transformation Kolmogorov–Smirnov test indicated that RT data in the MB and BB condition remained deviant from normal (SQRT transformation: *p* = 0.039 and *p* = 0.008; log/log 10 transformation, *p* = 0.052 and *p* = 0.026). Therefore, the non-parametric Friedman test for repeated measures was used to test for a difference in RT over the three conditions. As post hoc test the Wilcoxon Signed-Rank test was used. Effect sizes were calculated as *r* = *Z*/√*N* (Rosenthal 1994), with values 0.10—< 0.030 defined as being small, 0.30–0.50 as being medium and ≥ 0.50 as being large (Cohen 1977).

NeuroGuide (Version 3.0.0.1) with LE reference was used for generating tables of absolute power spectra of each individual for further analyses in SPSS (IBM SPSS Statistics for Macintosh, version 24.0). The power spectral value for any frequency intensity is: F(x) = (a^2^ (x) + b^2^ (x)). That is, the power spectrum is the sum of the squares of the sine and cosine coefficients at a specific frequency. A full description of the computation of the power spectrum can be found in Thatcher et al. (2007).

All variables were continuous and paired over the subjects, as all the subjects were exposed to all three conditions. To measure the effect of the auditory stimulation on the EEG, the absolute power in μV^2^ for each electrode for the frequencies 1–50 Hz was recorded and Linked Ears (LE) was chosen as reference for EEG analysis. Out of all recordings, the frequencies of 40 and 45 Hz of frontal electrodes F3, F4, F7, F8, Fp1, Fp2 and Fz as well as temporal electrodes T3, T4, T5 and T6 were chosen to focus on in this study. We choose to include the frequency of 45 Hz because auditory stimulation of 30–60 Hz induced maximal potentials around 45 Hz (Artieda et al. 2004) and visually evoked oscillations in the gamma band (40–48 Hz) have been found to reach values up to 46 Hz (Başar et al. 2015).

As electrodes T5 and T6 are also called parietal-temporal electrodes and have been renamed in the higher-resolution nomenclature (Modified Combinatorial Nomenclature; MCN) P7 and P8 (Oostenveld and Praamstra 2001), we selected these electrode locations to cover parietal measurements.

In addition, all data of the frontal electrodes were averaged and the same applies to the temporal electrodes.

As EEG data appeared to deviate from a normal distribution, the non-parametric Friedman test for repeated measures was used to test for a difference in μV^2^ over the three conditions. This test is the non-parametric alternative to the one-way ANOVA with repeated measures and provides the test statistic *χ*^2^, degrees of freedom and the significance level. Samples do not need to be normally distributed and dependent variables should be measured at the ordinal or continuous level. As this test does not allow for multivariate testing we repeated the Friedman test for each electrode, i.e., 11 tests were performed for 40 Hz and 11 tests for 45 Hz. In case of a significant effect, the Wilcoxon Signed-Rank test was used for post hoc testing. This test is the non-parametric equivalent to the dependent *t* test and provides a *Z* statistic and significance level.

We controlled for multiple comparisons of the frontal and temporal electrodes by applying Benjamini–Hochberg with a false discovery rate (FDR) of 0.20. This particular FDR was applied because hypothesis testing on power spectra changes in particular electrodes is quite exploratory and a higher FDR may avoid missing important results (McDonald 2014). The FDR can be applied in smaller studies and has the advantage to increase power when analysing multiple tests. The practical implications and benefits of applying an FDR level of 0.2 has been illustrated in real examples (Glickman et al. 2014).

To test whether results would be different by reducing multiple testing, we additionally performed Friedman tests for the averaged frontal and averaged temporal electrodes for 40 Hz and applied the same procedure for 45 Hz.

Bivariate Spearman correlations were calculated of RT and false responses on the Flanker task with the magnitude of the absolute power of the specific frequencies. Statistical significance was defined as *p* < 0.05. Tests concerning the results of the Flanker task were one-tailed.

## Results

**Fig. 1:**
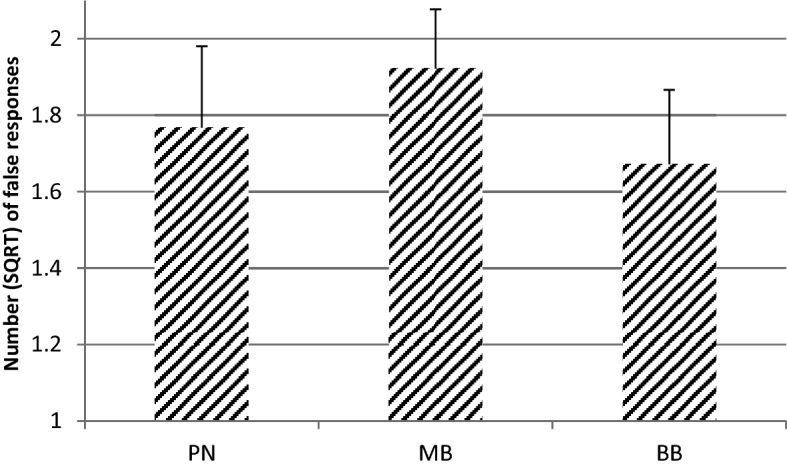
Mean (square root of) number (+ SE) of false responses on the Flanker task in PN, MB and BB condition (*n* = 24)

The data of the performance on the Flanker task was checked for outliers. One extreme outlier of > 4 SD was found concerning the number of false responses. This outlier was based on a number of 32 false responses in the MB and BB condition and 31 false responses in the PN condition. As the maximum number of false responses was 32 this participant likely had misunderstood the instruction. As a consequence, the data from this participant was excluded from data analysis. Results of the Friedman test indicated no significant differences in RT over the three conditions (*p* > 0.05). However, Anova indicated a significant difference between the PN, MB and BB condition for the number of false responses, *F* (2,42) = 5.972, *p* = 0.005, *partial η*^2^ = 0.221. Simple contrasts indicated a significant smaller number of false responses in the BB condition as compared to the PN condition, *F* (1,21) = 3, 486, *p* = 0.038, *partial η*^2^ = 0.142. In contrast, the number of false responses in the MB condition appeared to be larger than in the PN condition, *F* (1,21) = 18.711, *p* < 0.001, *partial* η^2^ = 0.471. A post hoc paired samples t-test indicated a significant smaller number of false responses in the BB condition than in the MB condition (*t* (23) = 1.78, *p* = 0.044, *partial* η^2^ = 0.122). No significant interaction between Gender and Condition was found (*p* > 0.05). Descriptive statistics are shown in Table 1 (RT) and Fig. 1 [number (SQRT) of false responses].

**Table 1:** Mean (M), standard deviation (SD) and median (Mdn) of reaction time (ms) on the Flanker task in the pink noise (PN), monaural beat (MB) and binaural beat (BB) condition (*n* = 24)

	PN			MB			BB		
	M	SD	Mdn	M	SD	Mdn	M	SD	Mdn
Reaction time (ms)	0.712	0.024	0.673	0.7108	0.019	0.702	0.733	0.035	0.686

For the EEG recordings data from 5 participants were excluded from data analysis due to noise in the recordings. The remaining data were analyzed for each frontal and temporal electrode. To establish the power to detect the hypothesized effects of the remaining 19 EEG datasets, we conducted a post-hoc power analysis using the program G* power 3.1.9.4 (Faul et al. 2007). After applying an effect size (eta)η^2^ = 0.03 (similar to *f* = 0.17, correlation = 0.85 and 3 conditions (PN, BB and MB), the obtained power was 0.82.

For the frequency of 40 Hz in the Fz electrode the Friedman test showed a significant difference, *Χ*^*2*^(2) = 8.444, *p* < 0.05. After controlling for the FDR by the Benjamini–Hochberg procedure with a false discovery rate of 0.2 for 7 frontal electrodes this difference remained significant. A post hoc test Wilcoxon Signed-Rank test indicated that the absolute power of 40 Hz in electrode Fz for the MB condition was significantly higher than for the PN condition, *Z* =  − 2.635, *p* < 0.01, *r* = 0.59. The test also showed that the absolute power for the condition MB was higher than the absolute power for the BB condition, *Z* =  − 2.678, *p* < 0.01, *r* = 0.60.

**Fig. 2:**
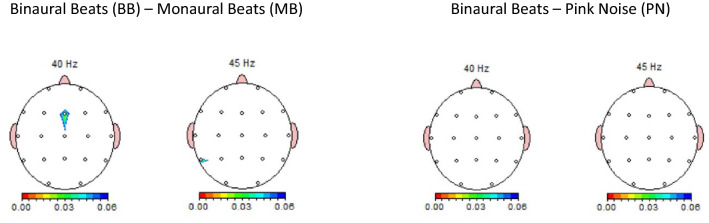
Topoplots of differences in absolute power between the BB and MB condition and between BB and PN condition

For 45 Hz in electrode T5 the Friedman test showed a significant difference, *Χ*^*2*^(2) = 7.111, *p* < 0.05. After controlling for the FDR by the Benjamini–Hochberg procedure with a false discovery rate of 0.2 for 4 temporal electrodes this difference remained significant. A post hoc test Wilcoxon Signed-Ranks test indicated that the absolute power of 45 Hz in electrode T5 for the BB condition was significantly higher than for the MB condition, *Z* =  − 2.243, *p* < 0.05, *r* = 0.50. A summary of the Friedman test results for electrodes 40 Hz and 45 Hz is shown in Tables 2, 3. In addition, topoplots of differences in absolute power between the BB and MB condition and between BB and PN condition are shown in Fig. 2. The first topoplot shows the significantly higher absolute power in the MB condition as compared to the BB condition.

**Table 2:** Friedman test results of absolute power (μV2) of separate electrodes for 40 Hz (*n* = 19)

	PN			MB			BB			*p*
	*M*	SD	Mdn	*M*	SD	Mdn	*M*	SD	Mdn	
F3	0.224	0.179	0.186	0.247	0.159	0.217	0.176	0.084	0.158	0.249
F4	0.247	0.324	0.179	0.245	0.150	0.209	0.200	0.135	0.174	0.211
F7	0.715	1.321	0.341	0.709	1.419	0.337	0.674	1.585	0.213	0.348
F8	0.755	1.587	0.272	0.741	1.374	0.333	0.724	1.572	0.271	1.000
Fp1	0.257	0.159	0.210	0.268	0.193	0.237	0.237	0.170	0.172	0.411
Fp2	0.372	0.417	0.241	0.487	0.759	0.282	0.294	0.524	0.167	0.066
Fz	0.183	0.101	0.160	0.232	0.146	0.176	0.166	0.070	0.160	**0.015**
T3	0.513	0.952	1.040	0.620	2.502	7.603	0.628	1.222	1.917	0.604
T4	0.946	0.961	0.720	0.802	2.018	4.034	0.633	0.978	0.904	0.728
T5	0.801	0.643	0.515	0.545	0.657	0.630	0.369	0.575	0.377	0.452
T6	0.846	0.756	0.547	0.610	0.815	0.835	0.520	0.674	0.525	0.571

*PN* pink noise, *MB* monaural beats, *BB* binaural beats, *M* mean, *SD* standard deviation, *Mdn* median

Benjamini–Hochberg significant *p* value is shown in bold

**Table 3:** Friedman test results of absolute power (μV2) of separate electrodes for 45 Hz (*n* = 19)

	PN			MB			BB			*p*
	*M*	SD	Mdn	*M*	SD	Mdn	*M*	SD	Mdn	
F3	0.234	0.195	0.173	0.207	0.137	0.172	0.195	0.146	0.158	0.678
F4	0.279	0.440	0.136	0.184	0.117	0.152	0.206	0.162	0.154	0.411
F7	0.687	1.340	0.347	0.724	1.360	0.295	0.556	1.128	0.202	0.348
F8	0.639	1.238	0.267	0.671	1.395	0.245	0.686	1.579	0.228	0.846
FP1	0.415	0.501	0.204	0.278	0.248	0.199	0.358	0.520	0.188	0.066
FP2	0.344	0.366	0.228	0.331	0.448	0.168	0.285	0.440	0.169	0.092
Fz	0.201	0.150	0.112	0.172	0.109	0.122	0.181	0.112	0.161	0.801
T3	1.075	1.200	0.731	2.865	9.207	0.576	1.146	1.835	0.535	0.801
T4	0.927	0.666	0.867	1.716	3.960	0.478	0.911	0.842	0.633	0.348
T5	0.585	0.436	0.450	0.573	0.713	0.350	0.645	0.506	0.498	**0.029**
T6	0.674	0.509	0.429	0.661	0.635	0.401	0.565	0.387	0.539	0.678

*PN* pink noise, *MB* monaural beats, *BB* binaural beats, *M* mean, *SD* standard deviation, *Mdn* median

Benjamini–Hochberg significant *p* value is shown in bold

Additionally, the averaged power of frontal and temporal electrodes was compared between the PN, MB and BB conditions. Neither significant difference was found for the frontal nor for the temporal electrodes. The results of the analyses are shown in Table 4. Finally, Spearman’s correlations were calculated within each condition to assess the relationship between the RT as well as number of false responses of the Flanker task and the total absolute power of 40 Hz and 45 Hz EEG frequencies. In the MB condition positive correlations were found between the RT and 40 as well as 45 Hz absolute power, indicating that higher power was associated with a longer RT. In addition, in the MB as well as BB condition higher power in the 40 as well as 45 Hz frequencies was associated with less false responses. No significant correlations were found in the PN condition. The results of the Spearman correlations are shown in Table 5.

**Table 4:** Non-parametric Friedman test results of the absolute power (μV2) of averaged frontal and averaged temporal electrodes under the pink noise (*PN*), monaural beat (*MB*) and binaural beat (*BB*) condition (*n* = 19)

	Hz	*p*	PN			MB			BB		
			M	SD	Mdn	*M*	SD	Mdn	*M*	SD	Mdn
Frontal	40	0.494	0.404	0.563	0.261	0.428	0.495	0.317	0.360	0.574	0.187
	45	0.291	0.412	0.541	0.293	0.372	0.487	0.239	0.360	0.500	0.204
Temporal	40	0.801	0.828	0.530	0.658	1.498	3.099	0.672	0.862	0.777	0.724
	45	0.801	0.815	0.555	0.711	1.454	3.579	0.577	0.817	0.725	0.651

*Hz* hertz, *M* mean, *SD* standard deviation, *Mdn* median

**Table 5:** Spearman correlations of the total absolute power (μV2) and performance (RT and number of false responses) on the Flanker task under the pink noise (*PN*), monaural beat (*MB*) and binaural beat (*BB*) condition (*n* = 19)

	Reaction time				False responses			
	40 Hz		45 HZ		40 Hz		45 Hz	
	*r*_*s*_	*p*	*r*_*s*_	*p*	*r*_*s*_	*p*	*r*_*s*_	*p*
PN	− 0.009	0.355	0.096	0.191	0.036	0.626	0.049	0.509
MB	0.184	**0.012**	0.177	**0.015**	− 0.250	**0.001**	− 0.242	**0.001**
BB	0.100	0.174	0.094	0.201	− 0.151	**0.039**	− 0.156	**0.033**

*Hz* hertz

Significant *p* values are in bold

## Discussion

In this study, the effects of BB and MB on RT and number of false responses on a Flanker task were measured while recording EEG. We expected that in particular BB would improve the performance on the attention task and would induce more absolute power of 40 or 45 Hz frequencies in the frontal and temporal electrodes. Thus, if BB indeed improves the performance on the Flanker task, a simultaneous increase of gamma power would indicate that the attention improvement might be the result of the BB-induced increase of gamma power. In the present study, the separate effects of the Flanker task performance and the BB on power spectra cannot be differentiated. However, the effects of the Flanker task itself on the power spectra can be assumed to be equal during the PN, MB and BB condition. As a consequence, we believe that the expected larger power during the BB condition can be attributed to the application of BB. However, although less likely, an improved task performance may in turn yield an additional increase in gamma power. In that case, increased gamma power may be the sum of task- and BB-induced gamma power.

With respect to attention, there was no difference in RT between any of the three conditions. However, the number of false responses in the BB condition was smaller than that in the PN condition while the number of false responses in the MB condition was larger than that in de PN condition. It appeared that the mean square root of false responses was largest in the MB condition, intermediate in the PN condition and smallest in the BB condition. The effect sizes of these differences were large, which can be considered to be quite substantial. Thus, in particular BB seems to increase attentional performance as compared to MB or PN. This finding is in line with the results of previous studies which found that beta and gamma BB improved the performance of vigilance and attention tasks (Colzato et al. 2017; Engelbregt et al. 2019; Lane et al. 1998). Notably, the designs of these studies were different. In the study of Lane et al. Colzato et al. and the present study the tasks were performed during the presentation of auditory stimuli whereas the tasks in the study of Engelbregt et al. were performed immediately after the presentation of the auditory stimuli. It may well be true that attention is more substantially improved during listening than after listening to BB. That would mean that the present effects of BB on attention are more pronounced because the Flanker task was performed during the presentation of BB. It may be recommended that future studies include a comparison of the direct and indirect effects of BB on attentional parameters.

According to our hypothesis, the BB-induced improvement on the Flanker task was expected to be accompanied by an increase in the absolute power of 40 or 45 Hz in frontal and/or temporal electrodes. However, against expectation, the 40 Hz absolute power of electrode Fz increased in the MB condition as opposed to PN and BB conditions. In addition, partly in line with our expectation the 45 Hz absolute power of electrode T5 increased in the BB condition relative to MB but did not differ from the PN condition. The present scarce and conflicting findings only justify the conclusion that BB has no consistent effect on EEG activity. Thus, we did not find convincing neurophysiological evidence that neural oscillation, i.e. neural synchronization, plays a role in BB-induced cognitive enhancement. This is in line with studies indicating that gamma BB do not enhance EEG spectral power in any frequency band or only induces a weak cortical entrainment (López-Caballero and Escera 2017; Perez et al. 2020). However, as is mentioned in the Introduction, the synchronization of fluctuations in gamma band power with intermittent and metrical auditory rhythms has been observed in MEG and EEG recordings (Fujioka et al. 2009; On et al. 2013). As the present cognitive enhancement could not be attributed to neural synchronization in the brain as reflected by an increase of 40–45 Hz power, the improved cognitive performance might be mediated by other factors. It has been proposed that BB enhances cognitive processing by the involvement of norepinephrine/glutamate dynamics, particularly the increase of phasic norepinephrine (Hommel et al. 2016; Mather et al. 2016). Although increased noradrenergic activity can be reflected by changes in the EEG, these changes may be observed in frequency bands other than we investigated. In a rat study, increased neuronal discharge activity of noradrenergic neurons of the locus coeruleus (LC) was found to alter forebrain and hippocampal electroencephalographic (EEG) activity. LC activation was consistently followed by high-frequency EEG activity (frequency bands 20.0–34.7 Hz and 34.7–43.8 Hz) in the frontal cortex and by the appearance of intense theta rhythm (2.7–6.8 Hz) in the hippocampus (Berridge and Foote 1991). Pharmacological studies and human brain imaging (MEG) or intracranial EEG studies in rats on theta and beta frequency band may be useful to further elucidate the effects of gamma BB.

To further explore any evidence of an association between performance on the Flanker task and EEG power measures we calculated the correlation between response parameters of the Flanker task and the total absolute power of 40 and 45 Hz EEG frequencies.

As gamma waves are associated with attentional selection, memory and associative learning (Teplan 2002), we expected that higher absolute power would be associated with a faster RT and a smaller number of false responses on the Flanker task. In line with our expectation, higher absolute power of 40 and 45 Hz EEG frequencies in the MB as well as BB condition appeared to be associated with a smaller number of false responses, meaning a better performance. However, we also found that a higher absolute power of 40 and 45 Hz in the MB condition was associated with a longer reaction time, reflecting worse performance. This finding is opposite to expectation. As a consequence, we may conclude that a better cognitive performance in the BB condition is associated with a higher 40 and 45 Hz power, whereas in the MB condition the better cognitive performance is accompanied by a slower reaction time. It must be noted that these higher absolute power in the MB or BB condition cannot be attributed to synchronization with ABS.

Summarizing, the present results indicate that BB can improve the quality of cognitive performance, in particular attention. As there was no association of BB with an increase in absolute 40 or 45 Hz power compared to PN or MB, the hypothesized neural entrainment in the brain, i.e., neural synchronization with gamma BB could not be confirmed by our EEG recordings. However, the present findings suggest that the quality of the performance of the Flanker task, at least in the BB condition, is positively related to the absolute gamma power. Thus, in spite of some evidence that cognitive performance seems to be associated with brain activity, as measured by EEG power, the cognitive enhancing effect of BB could not be explained by the notion of BB increasing the gamma power.

An explanation for the present controversial findings concerning the EEG recordings could be that the number of useable EEG recordings was quite low. A further limitation of the present study is the quite small and specific sample of students. It may well be true that the Flanker task is not sensitive enough for this highly educated sample, which may have caused Flanker RT parameters to deviate from a normal distribution. Furthermore, the minor heterogeneity of the sample might have caused that EEG power measures were also not normally distributed. As we, therefore, had to apply non-parametric tests more subtle differences between conditions could have been unnoticed. Finally, the present exposure of the participants to the auditory stimuli for 5 min could have been of a too short duration to induce major effects.

Based on the above-mentioned limitations we recommend that future research on BB should make use of a larger sample of participants to be sure that participants are a better representation of the overall population. Moreover, it could be preferred to study participants with a specific disorder, for example AD(H)D. Additional recommendations could be to focus on a broader range of cognitive functions to determine the effects of BB on the performance of other tasks. Finally, a better design could be to expose participants to the auditory stimuli for a longer time than 5 min as used in the present study.

Overall, the current findings show that listening to 40 Hz BB improves attention, as measured by the Flanker task. Although we found some evidence that the performance of the Flanker task was associated with a higher absolute power of 40 and 45 Hz frequencies, we could not confirm that BB improves attention by inducing a higher gamma power, reflecting the occurrence of neural entrainment.

## Data Availability

The datasets generated during and/or analyzed during the current study are available from the corresponding author on reasonable request.
